# ‘The baby will have the right beginning’: a qualitative study on mother and health worker views on point-of-care HIV birth testing across 10 sites in Zimbabwe

**DOI:** 10.1186/s12887-022-03601-x

**Published:** 2022-09-14

**Authors:** Emma Sacks, Leila Katirayi, Betsy Kaeberle, Haurovi William Mafaune, Addmore Chadambuka, Emmanuel Tachiwenyika, Tichaona Nyamundaya, Jennifer Cohn, Agnes Mahomva, Angela Mushavi

**Affiliations:** 1 Department of Global Health, George Washington University School of Public Health, 950 New Hampshire Ave, NW, Washington, DC, 20052 USA; 2 Elizabeth Glaser Pediatric AIDS Foundation, Washington DC, USA; 3 Elizabeth Glaser Pediatric AIDS Foundation, Harare, Zimbabwe; 4 Resolve to Save Lives, Geneva, Switzerland; 5 Ministry of Health and Child Care, Harare, Zimbabwe

**Keywords:** Birth testing, HIV/AIDS, Point of care, Postnatal care, Qualitative, Interviews, Zimbabwe

## Abstract

**Background:**

The survival of HIV-infected infants depends on early identification and initiation on effective treatment. HIV-exposed infants are tested at 6 weeks of age; however, testing for HIV sooner (e.g., shortly after birth) can identify in utero infection, which is associated with rapid progression. Infant early diagnostic virologic tests often have long turnaround times, reducing the utility of early testing. Point-of-care (POC) testing allows neonates born in health facilities to get results prior to discharge. This study aimed to understand the views of mothers and health workers regarding the use and acceptability of POC birth testing.

**Methods:**

Beginning in 2018, Zimbabwe offered standard HIV testing at birth to high-risk HIV-exposed infants; as part of a pilot program, at 10 selected hospitals, POC birth testing (BT) was offered to every HIV-exposed infant. In order to understand experiences at the selected sites, 48 interviews were held: 23 with mothers and 25 with health workers, including 6 nurses-in-charge. Participants were purposively sampled across the participating sites. Interviews were held in English, Shona, or Ndebele, and transcribed in English. Line-by-line coding was carried out, and the constant comparison method of analysis was used to identify key themes for each respondent type.

**Results:**

Findings were organized under four themes: challenges with BT, acceptability of BT, benefits of BT, and recommendations for BT programs. Overall, BT was well accepted by mothers and health workers because it encouraged mothers to better care for their uninfected newborns or initiate treatment more rapidly for infected infants. While the benefits were well understood, mothers felt there were some challenges, namely that they should be informed in advance about testing procedures and tested in a more private setting. Mothers and HCWs also recommended improving awareness of BT, both among health care workers and in the community in general, as well as ensuring that facilities are well-stocked with supplies and can deliver results in a timely way before scaling up programs.

**Conclusions:**

Mothers and health workers strongly support implementation and expansion of birth testing programs due to the benefits for newborns. The challenges noted should be taken as planning guidance, rather than reasons to delay or discontinue birth testing programs.

**Supplementary Information:**

The online version contains supplementary material available at 10.1186/s12887-022-03601-x.

## Patient and public involvement statement

The development of the research question and outcome measures were informed by previous research on patient priorities, experiences, and preferences. The direct outcome of the research is to assess patient preferences and acceptability of a new technology by asking patients (in the case of children: their families) and health care workers directly. However, patients were not involved in the design, recruitment, or conduct of the study. Findings will be shared with the Ministry of Health, who can share results with health facilities and patient populations. Participants are included in the acknowledgments.

## What is known about the subject

- Many HIV-exposed newborns are not tested for HIV or are tested too late to receive timely clinical intervention; long turnaround times for virologic tests compound the issue.
- Point-of-care HIV testing has been shown to greatly reduce turnaround time for infant HIV diagnostic tests at 6-8 weeks of age; point-of-care HIV testing for newborns offers the possibility of rapid testing and result return, often prior to hospital discharge after birth.
- There have been very few studies of birth testing, even fewer on point-of-care birth testing, and almost none on the use of point-of-care birth tests in routine settings across districts. Quantitative studies have suggested high uptake and effectiveness.
- Qualitative studies of early infant diagnosis have shown high acceptability among mothers and health care workers; however, some concerns have been raised about acceptability of birth testing and implementation of point-of-care testing in routine settings.

## What this study adds

- Point-of-care birth testing was well accepted by mothers and health workers in Zimbabwe because it encouraged mothers to better care for their uninfected newborns or initiate treatment more rapidly for infected infants.
- This study provides additional findings related to a maternal desire to be informed in advance about testing procedures and tested in a more private setting, as well as the need to ensure that facilities are well-stocked with supplies and community awareness of diagnostic options is high.
- Mothers and health workers strongly support implementation and expansion of birth testing programs due to the benefits for newborns. The study provides evidence to support the use of point-of-care birth testing in routine health care settings. The challenges noted should be taken as planning guidance, rather than reasons to delay or discontinue birth testing programs.

## Background

Prevention of mother-to-child HIV transmission has greatly reduced the number of children born with HIV, but in 2020, there were still 150,000 cases of perinatal transmission globally; despite greater availability of pediatric treatments, 37% of HIV-exposed infants are not tested in the first 2 months of life [1]. Mortality for untreated infants is high, with almost 50% succumbing to the disease before age two, with the highest risk occurring around 10 weeks of age [2]. Innovations in testing technologies and improved service delivery are urgently needed to address this gap.

Early infant diagnosis at age 6-8 weeks is recommended by the World Health Organization (WHO) and has been scaled up in many countries [3]. Where 6-week testing programs are strong, the WHO more recently recommended considering the addition of birth testing (BT) to the existing testing algorithm [4]. Because disease progression can be rapid among untreated infants, especially preterm or low birth weight infants, identifying infections immediately after birth and initiating a treatment regimen can greatly improve survival for the most at-risk infants [5].

Due to the need for virologic testing and lengthy laboratory processing procedures for infant blood samples, the turnaround times for conventional BT can be slow, reducing the clinical effectiveness of testing. Nonetheless, successful BT pilots and programs have been introduced in many countries in Southern Africa, including Eswatini, Malawi, Mozambique, South Africa, Zambia, and other countries [ 6, 7], although studies in each recorded challenges and delays, with some specifically noting the need for rapid on-site tests [8–11]. The introduction of point-of-care (POC) testing, which allows for on-site processing of blood tests, instead of sending samples to reference laboratories, provides the possibility to offer BT and receive results within hours or days, rather than weeks or months. Previous studies have shown that POC is feasible at birth [12, 13]; offering birth testing with POC with rapid availability of test results allows most mothers to know the result before discharge after delivery. Infants who are identified as HIV-positive can be enrolled in the HIV program per protocol and initiated on treatment as soon as possible.

Previous studies have shown that POC testing at age 6-8 weeks can reduce median turnaround time from sample collection to results from 55 to 0 days [14]. Building on the existing POC early infant diagnosis program in Zimbabwe, the Ministry of Health and Child Care worked with the Elizabeth Glaser Pediatric AIDS Foundation, with funding from Unitaid, to offer HIV testing at birth to every HIV-exposed infant born at or presenting to 10 hospitals throughout the country [15]. Studies around the time of data collection found an HIV prevalence among adults to range between 10 and 18% [16]; despite the high coverage of ART among pregnant women (92%), the prevalence of maternal-to-child transmission is still relatively high (8%) [17]. National data from Zimbabwe suggest that the proportion of HIV-exposed infants receiving a 6-8 week HIV test was 56% [18].

Implementation of BT and the use of POC tests requires both health care workers and mothers to find such testing acceptable [19]. Previous studies in Kenya and Eswatini found POC BT to be highly acceptable to mothers, as the reduction in waiting time for test results greatly reduced anxiety and fear. In these previous studies, although many families noted feeling concern about infant pain or fragility, this was noted as a concern but not strongly enough as to prevent acceptance of the test. POC BT was also found to be acceptable to health workers, although they also expressed concerns about health system capacity, workload of staff, sustainability of the program, and post-test engagement of patients in care [20, 21]. Due to the limited number of countries with point-of-care birth testing programs, and even fewer with POC BT offered in a routine health care setting, there is little previous qualitative evidence about its use and acceptability.

Following the introduction of POC BT in Zimbabwe, we carried out a qualitative study on the acceptability of POC BT among health care workers and mothers. This is one of the first studies to assess POC BT in a routine setting. This study was aimed at understanding the views of mothers and health care workers on POC birth testing in Zimbabwe, with the goal of understanding the necessary inputs for a successful POC birth testing program. It was part of a larger study of POC birth testing which also included quantitative data collection in the same 10 sites [14].

## Methods

Study hospitals (*n* = 10) were selected based on high volumes of births to HIV-infected women, and co-located maternities with an existing POC testing platform on site. Every infant born to an HIV-infected mother at or presenting with 72 h of birth to one of the 10 hospitals was eligible for testing. None of the hospitals previously had on-site laboratory testing, but all sites had the ability to send samples to the national reference lab for testing, if desired. Once POC was implemented, the use of non-POC was generally used only to resolve discrepant test results.

Qualitative participants were selected from the pool of mothers who were offered such a test, as well as health care workers in the same hospitals who were involved with BT. Most of the health care workers had experience with laboratory-based testing and could speak comparatively about the two types of tests after POC was introduced. Mothers may have had previous experiences with laboratory-based testing if they had given birth previously after receiving their HIV diagnosis; however, many mothers did not have this previous experience and therefore were not comparing but rather describing the experience of getting POC birth testing for their infant.

Recruitment for qualitative interviews took place over approximately a one-month period at each site. The maximum number and type of qualitative respondents was purposively chosen to include a range of stakeholders, namely health care workers and mothers, with potentially divergent experiences or views (See Table 1). Daily debriefings were held by members of the study team to determine if thematic saturation had been reached and no new themes were arising from interview participants.

**Table 1:** Maximum sample sizes for qualitative study participants

Study participants	Sample size	Assumptions and approximate number of participants per site
Study facilities	10 health facilities	10 health facilities with high numbers of HIV exposed infants identified per month were purposively selected to participate in the study
Heath care workers	Up to 30 health workers	Up to 3 health care workers at each facility
Health managers (lab or facility managers)	Up to 10 managers	Up to 10 managers across facilities
Mothers of HIV exposed infants	Up to 40 women	Up to 4 women per facility

Participants were approached to participate and consented by study staff and invited to participate regardless of their acceptance of testing or receipt of test result. Participants were informed that participation was fully voluntary and asked to provide written informed consent before beginning interviews. Interviews were held in private locations within the hospital facilities. Interviews were conducted by research assistants that were trained in study protocol procedures, conducting qualitative interviews, and human subject ethics, and who were supervised throughout the data collection process. Participants were given $5 to cover travel costs; health care workers were given $4 and a snack as a token of appreciation for their time.

Interviews were guided by pre-developed and pre-tested semi-structured field guides (See Supplemental Files). Interviews were held in English, Shona, or Ndebele, by multi-lingual study staff, depending on comfort and capacity of the interviewee. Interviews lasted between 40 min and 1.5 h and were audio recorded with permission of participants. Audio recordings were transcribed verbatim by multi-lingual research assistants familiar with the project. Inadvertent disclosure of names or other potentially-identifying information by respondents was redacted during transcription. Shona and Ndebele transcripts were translated into English. Study team members reviewed a selection of transcripts to ensure quality.

Analysis was guided by a phenomenological approach, which focuses on the commonality of lived experiences within particular groups. Data were uploaded into and analyzed using MaxQDA software version 12.0. General codes developed a priori based on previous knowledge of the topic and study objectives were used in the preliminary analysis of transcripts after reading each for familiarity. More detailed codes were developed during the analysis process, as nuance emerged, and further themes were identified from the data. Thematic analysis and constant comparison methods were utilized to identify and categorize each theme, according to the type of respondent [22]. Subthemes were organized into each theme and reviewed by multiple members of the study team before being finalized. Changes to theme and subtheme organization were noted and recorded. Illustrative quotes were selected to represent each theme.

Our study team included medical doctors, epidemiologists, public health researchers, and policymakers, all with experience in the study and provision of maternal and pediatric HIV care, especially in under-resourced settings. We began this study with prior research knowledge, as well as anecdotal and experiential knowledge, that in many contexts there are very long delays for infant HIV results, which leads to delays in treatment initiation and poor outcomes for infected children. Most team members had experience related to early infant diagnosis, both with and without POC; some had experience in research or care related to birth testing, including the involvement in a similar introduction of POC BT and study in Eswatini.

## Findings

A total of 48 interviews were conducted with participants. Twenty-three in-depth interviews (IDIs) were conducted with mothers and 25 IDIs were conducted with health care workers (HCWs), including 6 who were nurses-in-charge. The mothers were aged 21 to 40 years. All of the respondents accepted testing for their infant and, for most, the infant being tested was not their first birth. Of the HCWs, most were nurse midwives with 1 to 10 years of experience in their current maternity and/or postnatal unit. Almost all HCWs reported being trained in both early infant diagnosis (EID) and POC EID. Findings were organized under four themes: challenges with BT, acceptability of BT, benefits of BT, and recommendations for BT programs.

### Acceptability of birth testing

All mothers appreciated the availability of BT, valuing the ability to go home knowing the child’s status rather than waiting for a prolonged period to receive test results, generally at least 6 weeks. For infants who received a positive diagnosis, mothers appreciated being able to get the child on appropriate treatment early based on the HIV birth status.

*“I’m just happy that they tested the baby and gave me the results there and there I didn’t have to wait for three months, so I’m very happy with that.”* (*Mother*)

Many HCWs felt that BT POC testing was more convenient than standard dried blood spot (DBS) testing, with quicker results, but several highlighted that the convenience was only possible with enabling factors such as sufficient staff, training, and the availability of birth testing resources.

*“The [BT] program was readily accepted because when it was introduced… it came with less burden than DBS because DBS is strenuous plus it has long turn around…”* (HCW)

Mothers generally trusted the infant test result, and most did not plan to get a confirmatory test elsewhere, but stated they would wait to have the child tested again at 6 weeks as instructed. This was especially true for mothers of infants who tested HIV-negative. As one HCW highlighted, if the infant tests positive, mothers may raise questions about the test’s accuracy more so than if the baby tests negative.

*“I’m going to wait for my six weeks visit [to test the baby again], for now I will just give my baby the [prophylactic] medication then they will repeat the test at six weeks as told.” (Mother)*

*“They (mothers) do accept [the test results],… but then that’s when questions are like: ‘my child has become positive; isn’t the baby having my antibodies?’ otherwise when the baby is negative there are no questions, but if positive they will ask…” (HCW)*

### Benefits of birth testing

Mothers explained that during birth testing they were provided information on how to care for the infant according to the received result, and with this knowledge, it encouraged them to properly care for their child.

*“I was happy with that (birth testing) because they will have intervened early by protecting it so that it does not show that the baby has been diagnosed with HIV or not, because they had explained that if the baby is HIV positive and give it the required medication then the baby will be as healthy as other babies you will not notice any difference. As a parent I will be knowing how to protect the baby, what type of food I should give the baby and giving medication on time.” (Mother)*

Both mothers and HCWs highlighted that when mothers knew their infant was HIV-negative at birth, it can motivate the mother to try to keep the infant HIV-negative by closely following care instructions including medication administration, returning for appointments, and proper feeding.

*“We had a challenge: we said exclusive breastfeeding so the mother was not sure whether the baby will test negative or positive so some were just doing mixed feeding but ever since we started POC birth testing the mothers will be eager to know what to do which will make their babies negative and they will be having that zeal so that their babies remain negative...” (HCW)*

Most mothers and HCWs said one of the main benefits of birth testing is the ability to initiate an HIV-positive infant on treatment early, rather than waiting until the usual early infant diagnostic test was performed at age 4-6 weeks.

*“If the baby is supposed to be on medication, we may do so early instead of waiting for 6 weeks. It may be too late to wait for 6 weeks, and the child would have been affected so much.” (Mother)*

### Challenges with birth testing

Many mothers and HCWs discussed challenges with information about birth testing being provided to mothers too late. Several mothers noted that they would have preferred to learn about birth testing in advance, such as during ANC visits, rather than at delivery.

*“… what we knew way back was that a child gets tested at 6 months so when you are told a new thing now at first you are a bit puzzled because you don’t know the effects because it’s like an immediate thing which has just come so your mind spins and you don’t know how to accept it and you don’t know what to do…”* (*Mother*)

Both mothers and HCWs stated that some mothers may need and/or want to first discuss testing the child with their husband or partner. Some respondents described situations where the partner was not available at the time of delivery as a reason that the newborn was not tested at birth.

*“There are some people who still have to get consent from the partner… it becomes a challenge now because the husband might not be available at the time you want to do the test and the mother would tell you I cannot do this without the father’s consent.”* (HCW)

*“… if you are married and if you are in a relationship whereby you communicate before you do anything, you inform each other like are you ok with it… it [birth testing] is one of those things.”* (*Mother*)

A few respondents commented that mothers may not be in a mental state to care for an HIV-positive newborn right after delivery, including needing to be ready to take home antiretroviral treatment (ART) medication at discharge. Both mothers and HCWs noted this as a possible disincentive to accept birth testing, but mothers generally thought it was not enough of a reason to not test.

“*When she [a mother] gives birth to a baby who test positive, … she has to go home with drugs for the baby. It becomes difficult for her to adjust when the baby has tested positive, instead of us giving her time to think over it and come back. … People are not getting time to decide when the baby is positive, they are given medication for the baby, no time to think about it, to decide whether to give the baby [the medications] or not. I think that’s one of the challenges the mother won’t be prepared psychologically.*” (HCW)

Several mothers were appreciative that the testing was done in a private location away from other patients, and many respondents commented on the importance of privacy. For one mother the fact that the testing was not conducted in a private location, prevented her from asking all her questions as she wanted to maintain privacy.

*“… I was happy that it was done privately right. Where we were, no one knew where I was going. …” (Mother)*

*“I couldn’t ask [all my questions] because there was no time and I wanted to maintain privacy since we were in the ward.” (Mother)*

While most mothers said testing the newborn and receiving the results took less than 2 h, HCWs raised concerns that some mothers may not be willing to wait the additional time it takes for testing for a variety of reasons, such as transportation schedules or impatient family members waiting for the mother.

*“They [mothers] are willing, but to wait for 1 h waiting for results they won’t be comfortable… they will wait because they don’t have a choice, but you will see that they are not happy waiting.” (HCW)*

HCWs also explained that the wait time for results could be over an hour if there were stock outs of necessary supplies, a backlog of other samples, or other challenges with the POC machine. HCWs felt these delays might hinder mothers’ confidence in the program or affect receipt of the child’s result if the mother or another caregiver could not wait and was unable to return to the facility.

*“There is a challenge at times there will [be] 3 or 4 kids who will need [birth testing] and older kids 6 weeks who will need testing so the machine will be one. People will take time waiting to be tested so some end up going away.” (HCW)*

While most HCWs were not concerned about testing a newborn, a few were worried that a newborn was too young to be tested. Among the HCWs who expressed concerns, most felt that knowing the newborn’s status was more advantageous and outweighed any concerns about the infant’s age and fragility. A couple of mothers noted that it was difficult to see their child pricked and hear them cry, but for these mothers knowing the newborn status right away was more important than the momentary discomfort.

*“There are none [risks] except I was hurt a bit when they pricked the baby and it cried when they were taking blood - but there was nothing bad about it though*.” (*Mother*)

### Recommendations for birth testing programs

Participants gave recommendations for health system, facility, and community levels. Several HCWs suggested including birth testing information as part of health education during ANC visits to help mothers plan for any additional time to wait for test results, process information, and ask questions, as well as allow mothers to discuss testing the child with their partners prior to delivery. One HCW suggested providing mothers with written information materials they could take home.

“*I think [information should be given] through health education of the mothers during the ANC because they really need to know what is going to happen.” (HCW)*

*“The partner can be involved when they come with their partners for the routine ANC visits and we talk to them we allow them to ask questions and by the time they deliver maybe the partner would actually agree because they have information.” (HCW)*

At the individual facility level, both mothers and HCWs stressed that birth testing needs to be discussed in a private location, not in open wards; however, HCWs stated that their facilities may need additional private spaces for counseling, testing, and providing results.

*“Our infrastructure remains a challenge… because when you want to do birth testing soon after delivery you cannot do it because our set up is very poor, there will be other patients in the same room…and we cannot divulge anything. …” (nurse-in-charge)*

In addition, HCWs recommended that all facility staff, in particular nurse midwives and other maternal/postnatal department staff, should be educated about birth testing so that any staff member can identify and inform mothers about birth testing.

*“… we also need to conduct teachings, workshops, tutorials so that every health worker knows that there is birth testing so that even if a client comes and asks I have heard about this, any health workers in that facility must be able to explain and give information to that client.” (HCW)*

HCWs were unified in their recommendations to ensure that BT programs maintain a consistent and adequate supply of birth testing materials, and most notably cartridges, to ensure that all newborns can be tested and tested in a timely manner.

*“The people who are providing the cartridges should be aware of the number of babies that are born every month, maybe an average at this hospital, so that maybe they give enough resources to cater for all of them.” (HCW)*

To avoid backlogs and reduce wait times, HCWs suggested providing facilities with more than one POC machine and/or providing POC machines to smaller facilities.

*“… we sometimes have too many specimens to do at one [time], those that need testing at birth and the other ones being tested at 6 weeks all to be done with one machine, … it becomes a very big burden such that you don’t finish running the specimens in single a day and some people will be waiting and they will be complaining you are taking too long to produce the results. ….” (nurse-in-charge)*

Several HCWs also suggested educating the general community about birth testing to increase mothers’ awareness and acceptance of birth testing.

*“I think there should be community talks, like health education, health workers going to community impacting knowledge about the benefits of birth testing …”* (HCW)

Overall, mothers and HCWs strongly recommended continuing and expanding birth testing programs due to the related benefits, such as early initiation. They noted the challenges as program aspects to be considered, not a reason to delay or discontinue testing programs.

## Discussion

Mothers and health care workers were overwhelmingly accepting of POC BT, even as concerns about the program were noted, especially around the need for improved information to patients. Mothers noted ongoing structural challenges with health systems, such as the lack of private space in hospitals in which to discuss tests and results, and noted the need for introducing the topic of birth testing and point-of-care both to a wider audience in the community, and at appointments prior to delivery. Mothers also worried about the age and fragility of newborns to have a blood draw; however, no respondents believed this outweighed the need and benefit of having the test. Further, most mothers were grateful to receive test results sooner in order to act sooner to prevent transmission in the postpartum period or initiate treatment as soon as possible.

Health care workers equally saw a benefit of being able to provide test results quickly, especially as they previously had challenges contacting patients after discharge. Health care workers agreed with the need to raise correct information and awareness about BT generally in the community, but also noted the need to ensure consistent supplies of equipment (namely, cartridges) before scaling up programs, in order to ensure that every patient offered a test was actually able to receive one and get results in the promised timely way. Considering the benefits, mothers and health care workers, including in-charge nurses, were in favor of continuing, expanding, and promoting POC programs, including offering BT.

Findings from this study suggest that, while scaling up POC BT programs, attention should be paid to the health system aspects that are necessary for its success: specifically, ensuring private areas for patient testing and discussion, ensuring sufficient numbers of trained staff to run tests, strengthening forecasting and supply chain systems for testing equipment, and improving the provision of information to patients. As much as possible, health care workers should include discussions about birth testing and point-of-care options in antenatal care visits and allowing mothers time (both antenatally and postnatally) to ask questions about the test and be reassured about its safety and minimal pain and invasiveness, especially for newborns.

In other studies, both with POC and not, BT has been found to be generally acceptable among mothers and HCWs [23, 24]. Similar to this study, other studies have found parental concern about pricking newborns [23, 24], but in general BT was considered to be in the best interest of the child and the benefits seen to outweigh the concerns. Similar to this study, a study in Kenya reported that POC BT can improve newborn care and reduce parental anxiety [25]. The same study also found concerns that a HIV-positive result at birth would impact parent-infant bonding and negatively impact care [25], but this was not raised by participants in the present study.

Compared to conventional birth testing, POC birth testing offers a unique opportunity to ensure that infants are tested and lab results returned more quickly. A recent study from Zambia which evaluated a conventional BT pilot, reported a median turnaround time of 53 days [11]; a pilot study of POC BT in Eswatini found a median turnaround time of 13 days [12]. Thus, even with intensive support to conventional systems, POC BT is likely to significantly improve turnaround times in most settings. For women delivering in health facilities, most will be able to receive the result before being discharged from the facility.

One of the main challenges, regardless of testing method, is ensuring that infants who test HIV-positive are linked to care in order to begin treatment. The conventional testing study in Zambia found that only a third of infants diagnosed with HIV were linked to care [11], but the POC BT study in Eswatini found 84% of infants testing positive were initiated on treatment [12]. A qualitative study on birth testing in Lesotho found that treatment acceptance did not appear to be different between mothers learning their child was infected at birth versus other time points [24], and it is likely that linkage to care depends more on availability of treatment services or clinics and interoperability of medical record systems than parental acceptance. Other studies have found that mothers are very motivated to ensure their child receives the necessary treatments after being diagnosed with HIV [12].

One of the initial concerns raised with BT was whether parents would return in the 6-8 week window period for the follow-up test as recommended once they had already received a birth test. Quantitative data from the same study as presented here found a 46% return for subsequent testing within 8 weeks after an initial POC negative birth test [26]. The conventional testing study in Eswatini found that return rates for the follow-up appointment dropped from 78 to 74% after the introduction of BT [23], but the POC birth testing study in Eswatini found a 91% return rate for infants at 6-8 weeks who tested negative at birth [12]. A POC birth testing study in Kenya also found that 92% returned at the six-week follow-up period [27]. Thus, it appears feasible to encourage and achieve high levels of follow up testing for those testing negative at birth, given proper guidance and information. Qualitative research may be helpful to identify the barriers still outstanding for those who did not return for follow up testing.

On the health system side, this study identified the challenge of adequate staff and supplies, which has been reported elsewhere in the literature [25]. Challenges with stockouts, broken machines, expensive cartridges, and electrical blackouts remain persistent barriers to sustainable implementation. A study in Kenya comparing piloting POC machines against conventional lab-based processing found higher rates of missed testing opportunities, largely due to machine/stock outs and invalid results [27]. Thus, introducing new diagnostic options must be accompanied by standardized systems for forecasting and ordering supplies. Findings also emphasize that, despite the recognition of the importance of education, privacy, and community awareness, these activities are often not implemented well, if at all.

This study provides important insight into the experiences with POC BT but has some limitations. As this was not a longitudinal or comparative study, participants were not asked to directly compare laboratory-based to POC testing, nor birth testing directly to early infant (6-week) HIV testing. Health care workers spoke frequently about the advantages of POC over laboratory-based testing, as many had experience in both. Most mothers in the study only had the experience of POC BT, unless they had other children who were tested, most likely receiving conventional testing at 6 weeks, and possibly at different clinics under different circumstances. As it was possible to have a conventional BT or a 6-week POC test, the experiences of POC and BT together may be conflated. Although efforts were made to include a selection of participants from across multiple regions in the country, the selected facilities were only hospital level, and patients at other types of medical facilities (where the program might expand to) may be different. Further, future research would benefit from interviews with ministerial-level policymakers and administrators about potential challenges at the national or regional level. Lastly, because acceptance of BT was so high, we were unable to interview mothers who refused testing, and this minority of mothers may have differing views than the mothers who accepted testing. This is an important group, as they are the most vulnerable to not only poor clinical outcomes, but also social isolation; future studies may need different, targeted methods to gain perspectives from these families.

Because BT is so new, and POC BT even newer, this study provides important information about its acceptability among various stakeholders. Unlike previous pilot studies, this study’s strengths included collecting data at 10 hospitals in a wide range of regions, providing further confidence in the near-universality of acceptability, which echoes findings about POC in general. The study also provides one of the first analyses of acceptability of birth testing in a routine (non-study) setting, and as offered to every exposed dyad, rather than only those considered at highest risk of mother-to-child transmission. While there is consensus around the need to offer HIV testing as early as possible to those exposed, the long-term clinical advantage of early testing and treatment will need to be examined separately.

As POC BT is introduced in a country or region, the specific needs of the mothers and health care workers should be taken into consideration, as there may be differences in the experiences and concerns across countries. Future research should also seek to understand the views of the general community on HIV and birth testing and to identify the best strategies for raising community awareness and providing accurate information about new diagnostic options. Programs will need to implement strategies to improve linkage to care for infected infants as well as follow-up for subsequent testing for infants initially testing negative [28], as well as ensure consistent availability of supplies. As POC BT becomes available in lower-volume clinics and health facilities, further implementation research will be needed about how to ensure its optimal use and effectiveness.

## Conclusions

POC BT not only improves timeliness and clinical outcomes, but it is overwhelmingly acceptable to mothers and health care workers. Mothers’ concerns did not deter from testing, and more rapid results allowed for more immediate health-promoting actions. Most had positive experiences and wanted to share knowledge about the ability of the test with their communities. HCWs noted the need to strengthen supply chains in order that those seeking care receive it, as well as improve information flow about available diagnostic options to patients. The use of POC BT should be scaled up wherever possible, to reach the most at-risk infants, and to improve their health and survival.

## Supplementary Information

**Additional file 1.**

## Data Availability

Tools are available upon reasonable request from the corresponding author. Data contain potentially identifiable information and cannot be shared outside of the study team; however summarized reports can be shared on request.

## Abbreviations

AIDS: Acquired Immunodeficiency Syndrome
ARV: Antiretrovirals
BT: Birth Testing
DBS: Dried Blood Spot
EID: Early Infant Diagnosis
HCW: Health Care Worker
HIV: Human Immunodeficiency Virus
IDI: In-depth interview
POC: Point of Care
WHO: World Health Organization
