# Supplementary material for: ‘The baby will have the right beginning’: a qualitative study on mother and health worker views on point-of-care HIV birth testing across 10 sites in Zimbabwe
Source: BMC Pediatr. 2022 Sep 14;22:546. doi: 10.1186/s12887-022-03601-x (PMC9472398; doi:10.1186/s12887-022-03601-x)
Supplement: Supplementary file 1 — Additional file 1. [file 12887_2022_3601_MOESM1_ESM.zip › Appendix 3_ In-depth interviews for potential beneficiaries.docx]

|  | **Prompt** | **Response** |
| --- | --- | --- |
| **Consent** | | |
|  | Did the Caregiver sign the informed consent? | - 1: Yes - 2: No ***End the Interview*** |
|  | Was the Caregiver offered a copy of the Consent form? | - 1: Yes - 2: No ***End the Interview*** |
|  | **Illustrative questions and prompts** | **Response** |
| 1. 1 | Tell me about any previous experiences with infant testing. | |
|  |  | |
|  | Would you have any concerns about testing a child younger than 6 weeks for HIV?   1. Probe about vulnerability of infant 2. Probe if trust the results 3. Probe about stigma if positive | |
|  | Is there any reason that you would prefer waiting until 6 weeks for an HIV test? | |
|  |  | |
|  | Do you think there are some mothers or families who might not want to test their babies at birth?   1. Probe for reasons why not (health system, cultural, community, etc) | |
|  |  | |
|  | What do you think could be done to address the above concerns? | |
|  |  | |
|  | If birth testing was offered at some facilities, but not all, would that be a reason to reason to go there?   1. Probe for delivery location reasons 2. Probe for avoidance of testing | |
|  |  | |
|  | If birth testing was not offered where you delivered your baby or the baby was born at home, would you be willing to go for a birth test within 3 days?   1. Probe why or why not 2. Probe for when they would go | |
|  |  | |
|  | If a baby was identified to be HIV positive at birth, would you have any concerns putting an infant on treatment younger than 6 weeks of age?   1. Probe about why 2. Probe what age would be acceptable 3. Probe what types of treatment would be ok 4. Probe about who would need to be disclosed to in order to give treatment | |
|  |  | |
|  | If a baby was identified to be negative at birth, would you be willing to have the baby tested again at 6 weeks?   1. Probe reasons why or why not | |
|  |  | |
|  | Do you think testing for HIV at birth is a good or bad idea overall?   1. Probe for why 2. Probe what would need to change for it to be a good idea | |
|  |  | |
|  | If you have any fears about HIV testing at birth, what do you think should be put in place to address your fears ? | |
| Response |  | |
|  | Do you have any other comments on HIV testing at birth? | |
| Response |  | |
|  | **Demographic questions** | **Response** |
|  | Your age |  |
|  | How Many children do you have |  |
|  | Is the baby’s father aware of your HIV status? | - 1:Yes - 2: No - 99: Do not wish to answer |
| 1. A | Are all of the family members that you live with aware of your HIV status? | - 1:Yes - 2: No - 99: Do not wish to answer |
|  | Have any of your children received HIV testing | - 1:Yes - 2: No - 99: Do not wish to answer |
|  | Other than your visit today, have you been to this facility before? | - 1:Yes - 2: No - 99: Do not wish to answer |
|  | Overall, do you believe the health workers at this facility provide high quality services? | - 1:Yes - 2: No - 99: Do not wish to answer |
|  | Do you trust the health workers to keep your health information, including HIV status, confidential? | - 1:Yes - 2: No - 3: Not sure - 99: Do not wish to answer |
|  | If you are offered HIV testing for your child at birth how likely are you to accept to have your child tested  ***Rate yourself on a scale of 0-5 where 0 is not likely to accept and 5 being most likely*** | 0 1 2 3 4 5 |
|  | Can you explain your rating | |
| Response |  | |
