# Supplementary material for: ‘The baby will have the right beginning’: a qualitative study on mother and health worker views on point-of-care HIV birth testing across 10 sites in Zimbabwe
Source: BMC Pediatr. 2022 Sep 14;22:546. doi: 10.1186/s12887-022-03601-x (PMC9472398; doi:10.1186/s12887-022-03601-x)
Supplement: Supplementary file 1 — Additional file 1. [file 12887_2022_3601_MOESM1_ESM.zip › Appendix 4_ Indepth interviews with health workers in maternity.docx]

|  | **Prompt** | **Response** |
| --- | --- | --- |
| **Consent** | | |
|  | Did the health worker sign the informed consent | - 1: Yes - 2: No ***End the Interview*** |
|  | Was the Health worker given a signed Consent form | - 1: Yes - 2: No ***End the Interview*** |
| **Demographic Characteristics** | | |
|  | Profession | - 1: Nurse Midwife - 2: Nurse None Midwife - 3: Primary Counselor - 99: Other (specify |
|  | How long have been working in the maternity department | - 1: less than one year - 2: 1 to 2 years - 3: Above 2 years |
| **Trainings and confidence in Practicing POC birth testing** | | |
|  | Have you ever received any training in early infant diagnosis (EID) | - 1:Yes - 2:No |
|  | If yes did the training included POC EID | - 1:Yes - 2:No |
|  | Given your experience and training in Early Infant Diagnosis how do you rank your capacity to Conduct a DBS or POC to an new born infants (under 72 hrs. of life) on a scale of 0 to 5 ***where 0 means not confident at all and 5 means very confident*** | 0 1 2 3 4 5 |
|  | Can you explain why you gave yourself the rate you stated above? | |
| **Response** |  | |
| **Trainings and confidence in Practicing POC birth testing** | | |
|  | Have you ever received any training in Pediatric ART |  |
|  | Given your experience and training in Pediatric ART how do you rank your confidence to Initiate a new born infants (under 72 hrs. of life)on ART, rank yourself on a scale of 0 to 5 ***where 0 means not confident at all and 5 means very confident*** | 0 1 2 3 4 5 |
|  | Can you explain why you gave yourself the rate you stated above | |
| **Response** |  | |
|  | What kind of support do you think will useful to enable health workers to implement POC birth testing | |
| **Response** |  | |
| **Perceived Facilitators and Barriers for POC birth testing** | | |
|  | Given that the Ministry of health would want to roll out POC birth testing what do you think are the enablers for successful implementation of birth testing?  *(Client related/ Community related/ health systems related)* | |
| **Respons**e | 1. Client related 2. Community related 3. Health systems related | |
|  | Given that the Ministry of health would want to roll out POC birth testing what do you think are the barriers for successful implementation of birth testing?  *(Client related/ Community related/ health systems related)* | |
| **Response** | 1. Client related 2. Community related 3. Health systems related | |
|  | In view of the barriers above what do you think can be done to address these barriers and ensure successful role out? | |
| **Response** | 1. Client related 2. Community related 3. Health systems related | |
| **Health worker attitude** | | |
|  | Given your experience and the prevailing situation how do you rank the likelihood of success in implementing birth testing on a scale of 0 to 5 where 0 means the implementation is not likely to be a success and 5 means the program is most likely to succeed? | 0 1 2 3 4 5 |
|  | Can you explain why you gave yourself the rate you stated above | |
| **Response** |  | |
|  | Do you have any comments or advise on implementation of POC birth testing in health facilities in Zimbabwe? | |
| **Response** |  | |

***Thank you for your time***
