# Supplementary material for: ‘The baby will have the right beginning’: a qualitative study on mother and health worker views on point-of-care HIV birth testing across 10 sites in Zimbabwe
Source: BMC Pediatr. 2022 Sep 14;22:546. doi: 10.1186/s12887-022-03601-x (PMC9472398; doi:10.1186/s12887-022-03601-x)
Supplement: Supplementary file 1 — Additional file 1. [file 12887_2022_3601_MOESM1_ESM.zip › Appendix 5_Key informant interviews with Health managers.docx]

|  | **Prompt** | **Response** |
| --- | --- | --- |
| **Consent** | | |
|  | Did the Respondent sign the informed consent | - 1: Yes - 2: No ***End the Interview*** |
|  | Was the Respondent given a signed Consent form | - 1: Yes - 2: No ***End the Interview*** |
|  | | |
|  | Designation |  |
|  | Role in the Pediatric HIV program management |  |
|  | Do you think the Ministry of health and child care is ready to start implementing HIV testing at birth in public health facilities in the country | - 1:Yes - 2:No |
|  | Explain your answer above |  |
|  | What do you think are the key benefits of introducing Point of Care Birth testing at public health facilities in Zimbabwe |  |
|  | What do you think are the key strengths and opportunities within the health system which can facilitate the implementation of Point of Care Birth testing in Zimbabwe  *Probe for:*   1. *Health system related (policy, Human resources etc.)* 2. *Client related* 3. *Community related* 4. *Other general factors* |  |
|  | What do you think are the key weakness and threats to successful implementation of Point of Care birth testing in Zimbabwe  *Probe for:*   1. *Health system related (policy, Human resources etc.)* 2. *Client related* 3. *Community related* 4. *Other general factors* |  |
|  | What do you think should be done to address the challenges raised above |  |
|  | Do you have any comments regarding implementation of POC birth testing in Zimbabwe |  |

***Thank you for your time***
