# Supplementary material for: ‘The baby will have the right beginning’: a qualitative study on mother and health worker views on point-of-care HIV birth testing across 10 sites in Zimbabwe
Source: BMC Pediatr. 2022 Sep 14;22:546. doi: 10.1186/s12887-022-03601-x (PMC9472398; doi:10.1186/s12887-022-03601-x)
Supplement: Supplementary file 1 — Additional file 1. [file 12887_2022_3601_MOESM1_ESM.zip › Appendix 6_Indepth Interviews mothers after testing.docx]

|  | **Prompt** | **Response** |
| --- | --- | --- |
| **Consent** | | |
|  | Did the Caregiver sign the informed consent | - 1: Yes - 2: No ***End the Interview*** |
|  | Was the Care giver given a signed Consent form | - 1: Yes - 2: No ***End the Interview*** |
| **HIV testing** | | |
|  | What did you know about HIV testing for infants before the child was born?  ***Probe:*** *Please tell me some of the messages you had heard. When did you plan to have the child tested?* |  |
|  | Was the child born at home or at a facility?  ***Probe:*** *If born at the facility, were you given guidance about when to test the child for HIV?*  ***Probe:*** *If home, how did you know when to bring the baby in? Did you face any discrimination at the facility during a postnatal visit because of having a home birth?* |  |
|  | How did you feel about testing your baby for HIV at birth?  ***Probe:*** *was it your decision to test the baby?*  ***Probe:*** *Did you feel that it was important to test the baby? Why? Why not?* |  |
|  | Can you describe the experience of getting an HIV test for your baby?  ***Probe:*** *Do you feel like you received enough information about the test?*  ***Probe:*** *What additional information would you have liked to have received?* |  |
|  | Was the procedure well explained?  ***Probe:*** *were you comfortable with the procedure?*  ***Probe:*** *did you have additional concerns about testing that were not addressed?* |  |
|  | Do you you have any concerns about testing a child younger than 6 weeks for HIV?  ***Probe about vulnerability of infant***  ***Probe if trust the results***  ***Probe about stigma if positive*** |  |
|  | Is there any reason that you would prefer waiting until 6 weeks for an HIV test? |  |
|  | How long did you expect to wait to get the HIV results for the baby?  ***Probe:*** *how long did you wait to receive the results?*  ***Probe:*** *did the service meet expectations? Slower, faster?*  ***Probe:*** *if had to return for results: were you able to return when recommended? Why or why not? What would have been a reasonable time frame to return?* |  |
|  | Were you satisfied with how you and your baby were treated when your baby received its HIV test?  ***Probe:*** *why/ why not*  ***Probe:*** *Are certain groups ever treated differently when they come in for an infant HIV test? Rural vs. urban residents, women who delivered at home vs facility, rich vs. poor, married vs. unmarried, etc.* |  |
|  | Have you had previously had an HIV test for a different child?  ***Probe:*** *If yes, how did the experience compare? Same, better or worse*  ***Probe:*** *If yes, how long did it take to get results compared to previous test? Longer time, same time or shorter time* |  |
|  | Do you trust the results of the test? Why or why not?  ***Probe:*** *If not, what do you plan to do?*  ***Probe:*** *What would make you believe the results more? (example, to see the results; if the blood was sent to another facility first).* |  |
|  | Did you feel confident that the health care worker knew about infant testing?  ***Probe:*** confidence in knowledge, skill, honesty of health care worker |  |
|  | Was the child found to be HIV infected? | - 1:Yes - 2:No **skip to 401** - 3: Do not wish to disclose: **Skip to 401** |
| **Mothers with HIV infected Children** | | |
|  | Did you start the child on ART? Why or why not?  ***Probe:*** *If not, do you plan to?*  ***Probe:*** *If not, why not? (concerns about vulnerability, stigma, disclosure)* |  |
|  | How much information did you receive about why babies should be on ART?  ***Probe:*** *What are some of the messages you got?* |  |
|  | How easy do you think it will be to keep the baby on ART?  ***Probe:*** *Could you talk about some of the challenges that you worry you might experience?* |  |
|  | Will you come back to a facility?  ***Probe:*** *Which facility?*  ***Probe:*** *When and why?*  ***Probe:*** *did you get instructions about coming back for another test?* |  |
|  | How will you travel back to the clinic?  ***Probe:*** *issues in transport, cost, etc.* |  |
|  | What would make it easier to get tested?  ***Probe:*** *testing available more days/hours?*  ***Probe:*** *time in queue, time to get results*  ***Probe****: transport, cost (are you aware the test is free?)* |  |
|  | How could the health facility make testing an infant for HIV easier for the caregiver or infant?  ***Probe:*** *please try to think about three things* |  |
|  | Is there anything else you would like to tell us about your experience with infant HIV tests? |  |
|  | **Mother with children HIV negative** |  |
|  | Given that your child tested Negative how likely are you to come back for HIV testing at 6 weeks  ***Rate yourself on a scale of 0-5 where 0 is not likely to accept and 5 being most likely (circle your response)*** | 0 1 2 3 4 5 |
|  | Explain your rating for the above question |  |
|  | Do you have an exact date what you plan to return for testing? | - 1:Yes - 2:No - 3: Not sure |
|  | Do you plan to return to the same facility or different for another HIV test? | - 1:Same - 2:Different - 3: Not sure |
|  | Do you think that there are some women whose children tested negative at birth who are not likely to come back with their children for HIV testing at 6 weeks | - 1:Yes - 2:No - 3: Not sure |
|  | If yes what could be their reasons for not coming back |  |
|  | How could the health facility make testing an infant for HIV easier for the caregiver or infant?  ***Probe:*** *please try to think about three things* |  |
|  | Is there anything else you would like to tell us about your experience with infant HIV tests? |  |
| **General questions** | | |
|  | Do you think testing for HIV at birth is a good or bad idea overall?  ***Probe for why***  ***Probe what would need to change for it to be a good idea*** |  |
|  | Do you think there are some mothers or families who might not want to test their babies at birth?  ***Probe for reasons why not (health system, cultural, community, etc)*** |  |
|  | If birth testing was offered at some facilities, but not all, would that be a reason to reason to go there?  ***Probe for delivery location reasons***  ***Probe for avoidance of testing*** |  |
|  | What do you think could be done about these concerns? |  |

|  | **Demographic questions** | **Response** |
| --- | --- | --- |
|  | Your age |  |
|  | How Many children do you have |  |
|  | Is the baby’s father aware of your HIV status? | - 1:Yes - 2: No - 99: Not willing to disclose |
| 1. A | Are all of the family members that you live with aware of your HIV status? | - 1:Yes - 2: No - 99: Not willing to disclose |
|  | Have any of your children received HIV testing | - 1:Yes - 2: No - 99: Not willing to disclose |
|  | Other than your visit today, have you been to this facility before? | - 1:Yes - 2: No - 99: Not willing to disclose |
|  | Overall, do you believe the health workers at this facility provide high quality services? | - 1:Yes - 2: No - 99: Not willing to disclose |
|  | Do you trust the health workers to keep your health information, including HIV status, confidential? | - 1:Yes - 2: No - Not sure - 99: Not willing to disclose |
|  | Do you have any other comments on HIV testing at birth? | |
|  |  | |

***Thank you for your time***
